# PERK/CHOP contributes to the CGK733-induced vesicular calcium sequestration which is accompanied by non-apoptotic cell death

**DOI:** 10.18632/oncotarget.4487

**Published:** 2015-07-10

**Authors:** Yufeng Wang, Yasuhiro Kuramitsu, Byron Baron, Takao Kitagawa, Junko Akada, Kazuhiro Tokuda, Dan Cui, Kazuyuki Nakamura

**Affiliations:** ^1^ Department of Biochemistry and Functional Proteomics, Yamguchi University Graduate School of Medicine, Ube, Japan; ^2^ Department of Pathology, Yamguchi University Graduate School of Medicine, Ube, Japan; ^3^ Centre of Clinical Laboratories in Tokuyama Medical Association Hospital, Shunan, Japan

**Keywords:** CGK733, calcium sequestration, PERK/CHOP, ER stress, non-apoptotic death

## Abstract

Calcium ions (Ca^2+^) are indispensable for the physiology of organisms and the molecular regulation of cells. We observed that CGK733, a synthetic chemical substance, induced non-apoptotic cell death and stimulated reversible calcium sequestration by vesicles in pancreatic cancer cells. The endoplasmic reticulum (ER) stress eukaryotic translation initiation factor 2-alpha kinase 3/C/EBP homologous protein (PERK/CHOP) signaling pathway was shown to be activated by treatment with CGK733. Ionomycin, an ER stress drug and calcium ionophore, can activate PERK/CHOP signaling and accelerate CGK733-induced calcium sequestration. Knockdown of CHOP diminished CGK733-induced vesicular calcium sequestration, but had no effects on the cell death. Proteomic analysis demonstrated that the ER-located calcium-binding proteins, calumenin and protein S100-A11, were altered in CGK733-treated cells compared to non-treated controls. Our study reveals that CGK733-induced intracellular calcium sequestration is correlated with the PERK/CHOP signaling pathway and may also be involved in the dysregulations of calcium-binding proteins.

## INTRODUCTION

Calcium ions (Ca^2+^) are correlated with cellular life in almost all respects. Calcium signaling has been found to contribute to various life activities, such as muscle contraction, neuronal transmission, cellular motility, cell growth and proliferation and other biochemical roles include regulating enzyme activity and components of the cytoskeleton [1–3]. Intracellular calcium metabolism or homeostasis play decisive roles for cells, including gene expression, signal transduction, programmed cell death and autophagy [4, 5]. This important signaling molecule can exert allosteric regulatory effects on enzymes and proteins through their release into the cytoplasm [1]. To maintain the homeostasis of Ca^2+^ at an appropriate concentration in the cytoplasm (10–100 nM), Ca^2+^ is actively pumped from the cytosol to the extracellular space and into the endoplasmic reticulum (ER).^4^

The ER is correlated with many cellular processes, including maturation, folding, transport of protein and Ca^2+^ homeostasis, all of which are required for cell survival and normal cellular functions [6–8]. It serves as a dynamic pool of Ca^2+^, which facilitates Ca^2+^ movements within the cell by avoiding cytoplasmic routes and is involved in rapid signaling events associated with cell stimulation [9]. The ER stress triggers several forms of cellular stress responses and is intimately involved in apoptosis through alterations in Ca^2+^ homeostasis and depletion of the ER Ca^2+^ store from the ER lumen [10–13] Cytochrome c binds to inositol 1,4,5-trisphosphate receptor (IP3R) on the ER membrane in early apoptosis, resulting in calcium release into the cytoplasm, which stimulates a mass exodus of cytochrome c from the mitochondria that induces subsequent apoptotic cell death [14, 15]. C/EBP homologous protein (CHOP) is an important executor in ER stress-induced apoptosis through the ER oxidoreduclin-1α (ERO1α)/IP3R/Ca^2+^/calmodulin dependent protein kinase II (CaMKII) pathway and the B-cell lymphoma 2 (Bcl-2) family member pathway [16]. The released calcium, induced by CHOP, triggers the activation of CaMKII and subsequently activates downstream apoptotic signals, including signal transducers and activators of transcription (STAT1), jun amino-terminal kinases (JNK)-first apoptosis signal (fas), reactive oxygen species (ROS) and mitochondrial cytochrome c [17].

CGK733 has been reported to suppress cell survival of drug-induced senescent tumor cells and trigger the non-apoptotic death through suppression of cyclin D1 [18, 19]. Here, we investigate if CGK733 can trigger the ER stress and disrupt calcium homeostasis. We examined the behaviors of ER stress signals and calcium status as well as cytotoxicity in response to CGK733 in pancreatic cancer cell lines. CGK733 was shown, as expected, to have remarkable effects on the ER stress and the cell death. Interestingly, however, CGK733 induced a cytoplasmic vesicular calcium sequestration and failed to trigger apoptosis.

## RESULTS

### CGK733 induces non-apoptotic death in pancreatic cancer cells

**Figure 1:**
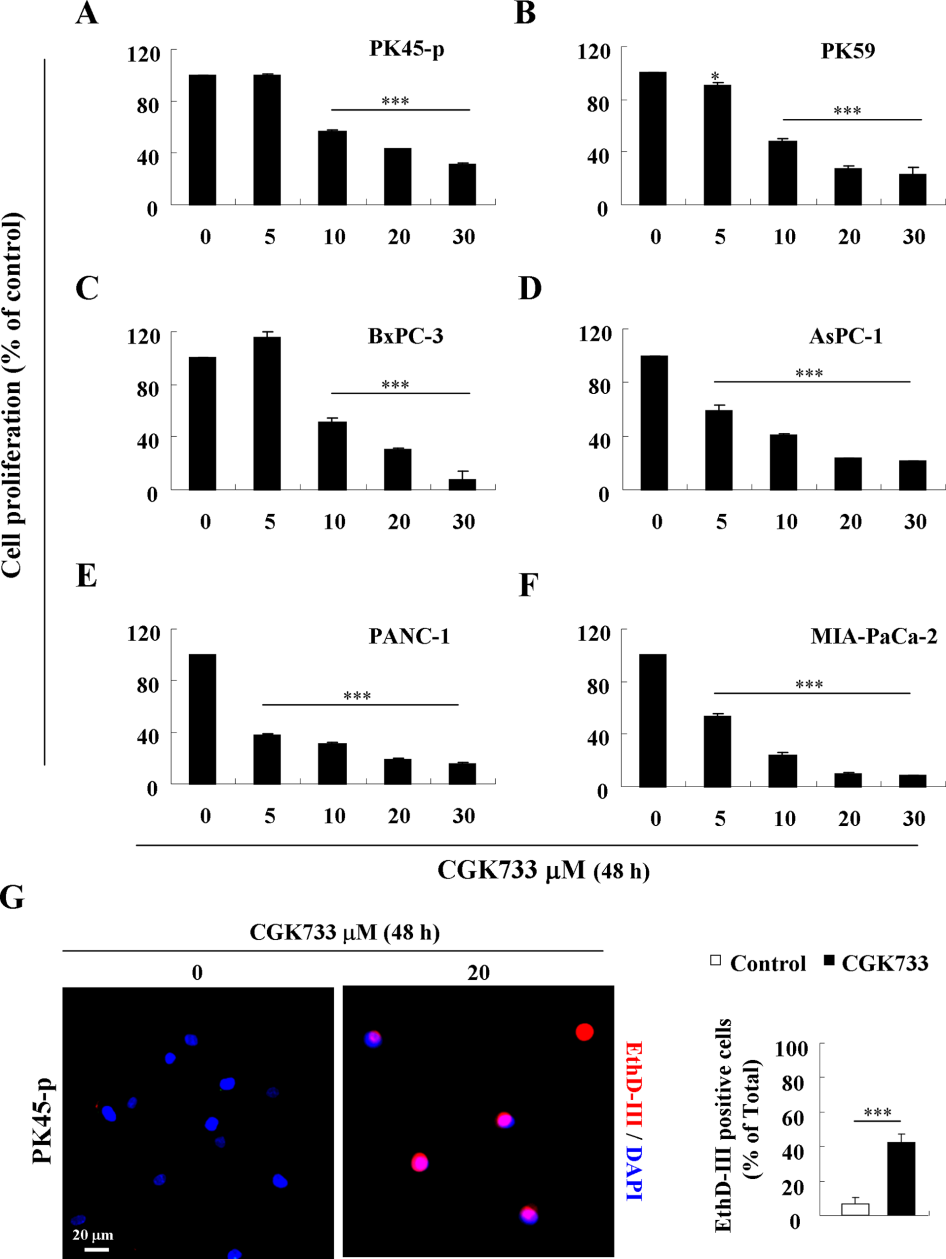
CGK733 induced cell death in pancreatic cancer **A–F.** Pancreatic cancer cell lines were treated with CGK733 for 48 h in a dose dependent manner. Viability was detected by MTS assay. **G.** PK45-p cells were stained by EthD-III (red) and DAPI (blue) after cells were treated with 20 μM of CGK733 for 24 h. The quantification is shown in the right panel. Bars, SD; *, *p* < 0.05; ***, *p* < 0.001.

Recently, CGK733 has been shown to induce cell death in breast, lung, and colon carcinoma cells by modulating ATM, p21 (CIP1) and cyclin D1 [18, 19]. We thus investigated the induction of cell death following 48 h of 20 μM CGK733 exposure, in six pancreatic cancer cells. Viability was shown to be significantly reduced by treatment with CGK733 in all the types of pancreatic cancer cell lines in a dose dependent manner (Figure 1A–1F). Ethidium homodimer III (EthD-III) fluorescent assay in PK45-p cells showed that more than 50% of cells were dead with intact nuclei and loss of cellular membrane (Figure 1G). As these results indicated that CGK733 induced cell death through an apoptosis-independent pathway, the caspase-dependent apoptotic pathway or the tumor necrosis factor alpha (TNF-α)-dependent necroptotic pathway was blocked by pre-treatment with a pan-caspase inhibitor (z-VAD-fmk) or a receptor-interacting protein 1 (RIP1) kinase inhibitor (Necrostatin-1), respectively. Although, CGK733 has been observed to trigger pro-apoptosis activity (caspase-3 cleavage, data not shown), neither inhibitor could rescue the death of PK45-p and PK59 cells from CGK733-induced cytotoxicity (Supplementary Figure S1). Taken together, these data indicated that CGK733-induced cell death in pancreatic cancer cells is through an apoptosis- or a necroptosis-independent pathway.

### CGK733 induces cytoplasmic vesiculation accompanied by cell death

**Figure 2:**
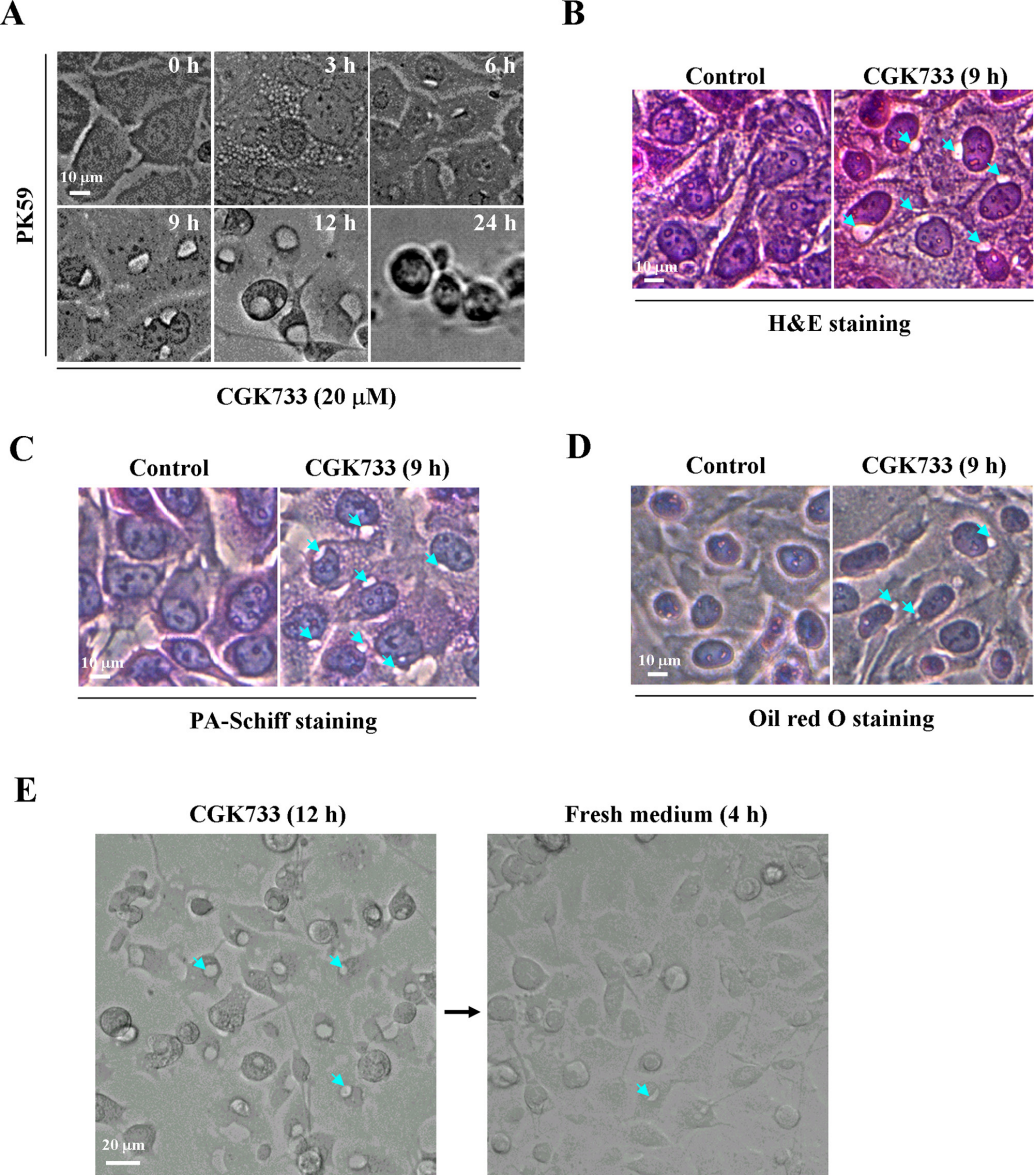
CGK733 induced reversible vesiculation in pancreatic cancer cells **A.** The vesicles were observed under a microscope over a time course, after PK59 cells were treated with 20 μM of CGK733. **B.** H&E staining was performed after PK59 cells were treated with 20 μM of CGK733 for 24 h. **C.** PA-Schiff staining was performed after PK59 cells were treated with 20 μM of CGK733 for 24 h. **D.** Oil red O staining was performed after PK59 cells were treated with 20 μM of CGK733 for 24 h. **E.** Cells were cultured in the absence of CGK733 for 4 h after cells were treated with 20 μM of CGK733 for 12 h. Arrows indicate the observed vesicles.

**Figure 3:**
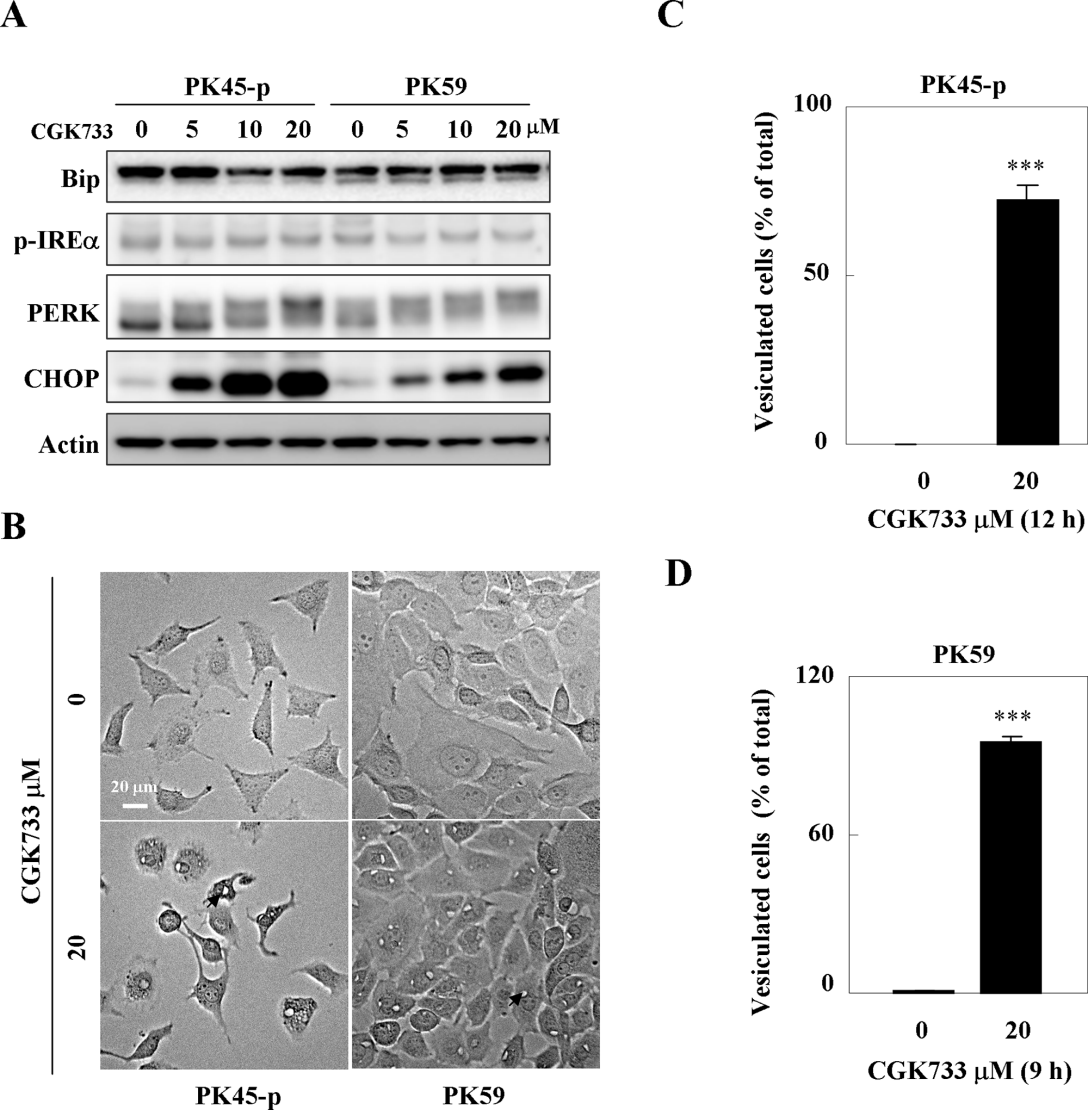
CGK733 induced ER stress through the PERK/CHOP signaling pathway **A.** The expression of ER stress markers were detected by Western blotting after cells were treated with CGK733 for 6 h in a dose dependent manner. **B.** The vesicles were observed under a microscope after PK45-p and PK59 cells were treated with 20 μM of CGK733 for 12 h and 9 h, respectively. **C.** and **D.** Vesiculated cells were quantified in CGK733-treated PK45-p and PK59 cells, respectively. Bars, SD; *** *p* < 0.001; arrows indicate the observed vesicles.

Following treatment of PK59 cells with 20 μM CGK733, vesicles were observed in the cytoplasm at 3 h, increasing in size at 6 h and reaching maximum size at 12 h, with loss of cytoplasm and death occurring after 24 h (Figure 2A). To identify the type of vesicle and its contents, cells exposed to CGK733 for 6 h were sucessfully stained with Hematoxylin and eosin (H&E) (Figure 3B), but not Periodic acid-Schiff (PAS) (Figure 3C) or oil red O (Figure 3D) staining, indicting that CGK733-induced vesiculation was not through intracellular glycogen storage or lipidosis. Moreover, vesiculation and cell death induced by CGK733 can be reversed within 4 h after drug withdrawal (Figure 2E and data not shown). These results demonstrated that CGK733 triggered reversible vesiculation (but not glycogen storage or lipidosis) which is accompanied by cell death in pancreatic cancer.

### CGK733 induces the ER stress response through PERK/CHOP

Treatment of PK45-p and PK59 cells with CGK733 for 6 h activated PERK and up-regulated its downstream target CHOP in a dose dependent manner (Figure 3A). However, CGK733 failed to elevate the expressions of phosphor (p)-IREα and Bip, indicating that the CGK733-induced ER stress response is dependent on the PERK/CHOP signaling pathway. Meanwhile, a mass vesiculation (an incidence of over 75%) was observed after PK45-p and PK59 cells were treated with 20 μM CGK733 for 12 h and 9 h respectively (Figure 3B; quantifications shown in Figure 3C and 3D). These results indicated that the CGK733-induced vesiculation possibly contributes to the ER stress response through the PERK/CHOP signaling pathway.

### Ionomycin enhances the CGK733-induced ER stress response and accelerates vesicular calcium sequestration

**Figure 4:**
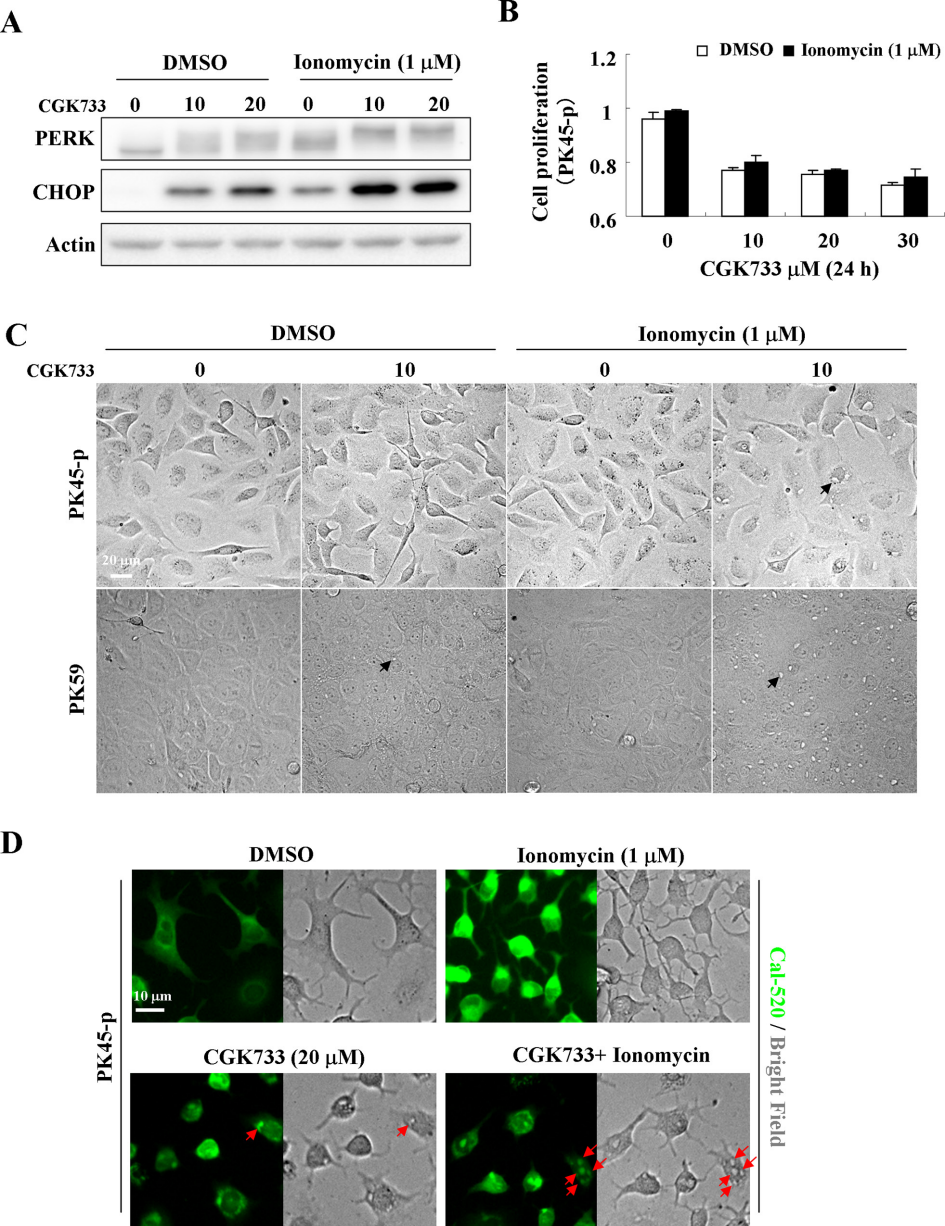
Ionomycin enhanced CGK733-induced PERK/CHOP activation and vesicular calcium sequestration **A.** The expression of PERK and CHOP were detected by Western blotting after cells were treated with CGK733 for 6 h in the presence or absence of 1 μM of ionomycin. **B.** MTS viability assays were performed after cells were treated with CGK733 for 24 h in the presence or absence of 1 μM of ionomycin. **C.** The vesicles were observed under a microscope after PK45-p and PK59 cells were treated with 10 μM of CGK733 in the presence or absence of 1 μM of ionomycin for 12 h and 9 h, respectively. **D.** Cal-520 fluorescence combined with bright field microscopy was performed after cells were exposed to 20 μM of CGK733 for 12 h in the presence or absence of 1 μM of ionomycin. Bars, SD; Black arrows indicate the observed vesicles; red arrows indicate the co-localization of calcium ions with the vesicles.

To understand the relationship between CGK733-induced vesiculation and the ER stress response, ionomycin, a calcium ionophore that causes ER stress through Ca^2+^ transport from the ER [20, 21] was used to enhance ER stress by CGK733 treatment (Figure 4A). PK45-p cells were pre-treated with 1 μM of ionomycin for 30 min, and then with CGK733 for 6 h, leading to an increase in PERK/CHOP signaling activity (Figure 4A). Vesiculation was remarkably increased in PK45-p and PK59 cells following treatment of 10 μM CGK733 combined with ionomycin compared to the treatment of CGK733 alone for 12 h and 9 h, respectively (Figure 4C). Moreover, ionomycin did not hasten cell death by CGK733 (Figure 4B), indicating that the CGK733-induced ER stress response is possibly distinguished from its induction of cell death. We then examined whether CGK733-induced vesiculation was the result of calcium accumulation. Expectedly, localization of calcium fluorescent signals (by Cal-520 fluorescent assay) match perfectly the vesicles produced by CGK733 treament (Figure 4D), indicating that CGK733-induced vesiculation resulted mainly from calcium sequestration. To clear the accumulated calcium sources, the extracellular or intracellular calcium was depleted by treatment with EGTA or Thapsigargin, respectively. We found that Thapsigargin but not EGTA could inhibit the CGK733-induced calcium sequestration in PK59 cells (Supplementary Figure S2), indicating that the accumulated calcium mainly derived from the intracellular pool. Taken together, these results revealed that CGK733 induced intracellular calcium sequestration by vesicles, which was accompanied by, but distinguished from, the consequent non-apoptotic cell death.

### Knockdown of CHOP diminished CGK733-induced calcium sequestration in vesicles

**Figure 5:**
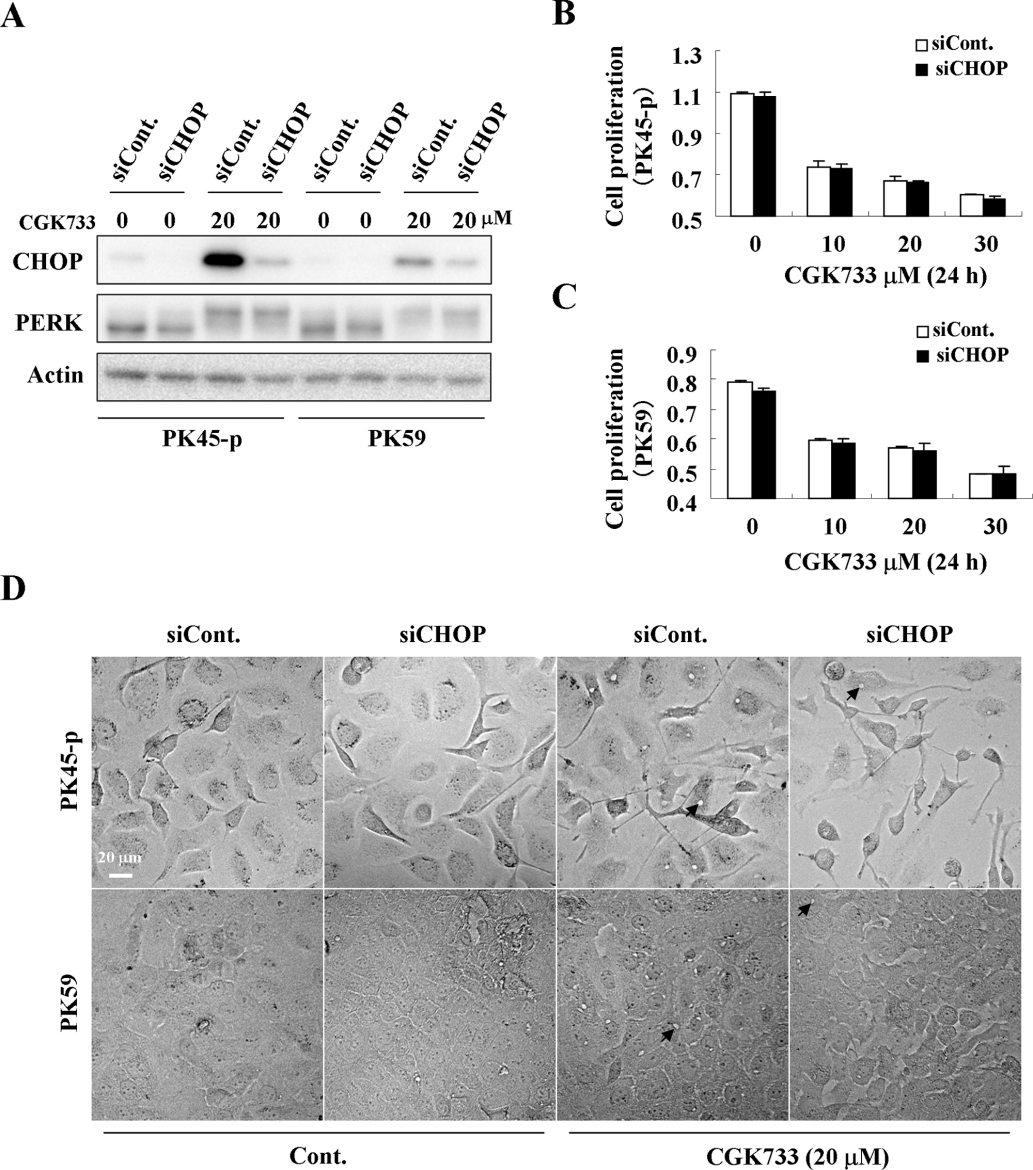
CHOP is involved in the calcium sequestration in vesicles **A.** Cells were treated with 20 μM of CGK733 for 6 h after knockdown of CHOP by siRNA for 48 h. **B.** and **C.** MTS viability assays were performed after cells were treated with CGK733 for 24 h after knockdown of CHOP by siRNA. **D.** The vesicles were observed under a microscope after PK45-p and PK59 cells were treated with 20 μM of CGK733 for 12 h and 9 h respectively after knockdown of CHOP by siRNA. Arrows indicate the observed vesicles.

To test whether the CGK733-induced vesicular calcium sequestration is correlated with the PERK/CHOP signaling pathway as a result of the ER stress response, we performed an siRNA knockdown assay against the CHOP gene in both PK45-p and PK59 cells (Figure 5A). Expectedly, knockdown of CHOP dramatically delayed CGK733-induced vesicle formation in both PK45-p and PK59 cells (Figure 5D). However, knockdown of CHOP did not rescue the cells from death induced by CGK733 treatment (Figure 5B and 5C), revealing that the expression of CHOP did not contribute to CGK733-induced non-apoptotic cell death. These results indicated that the PERK/CHOP signaling pathway is correlated with the vesicular calcium sequestration, but not contribute to the consequent non-apoptotic cell death induced by CGK733 treatment.

### Calumenin and protein S100-A11 were shown to be altered after cells were exposed to CGK733 by proteomic technology

**Figure 6:**
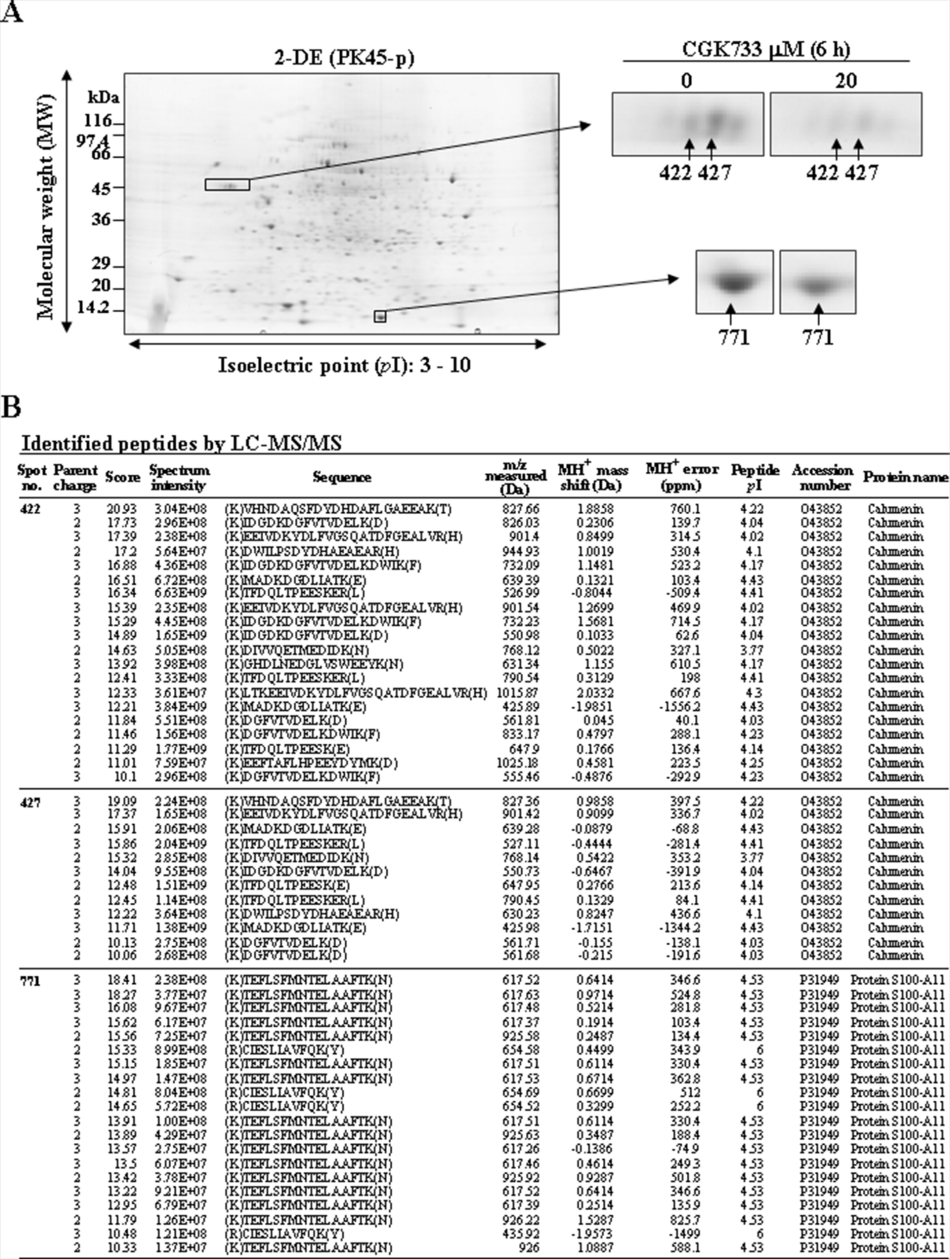
Calumenin and protein S100-A11 were identified and shown to be downregulated by treatment with CGK733 **A.** Three downregulated protein spots were identified in the 2-DE gel image of the CGK733-treated cell group compared to the control group. **B.** MS analysis was performed by the Agilent Spectrum Mill MS proteomics workbench against the Swiss-Prot protein database search engine. The protein numbers are same as in (A).

**Figure 7:**
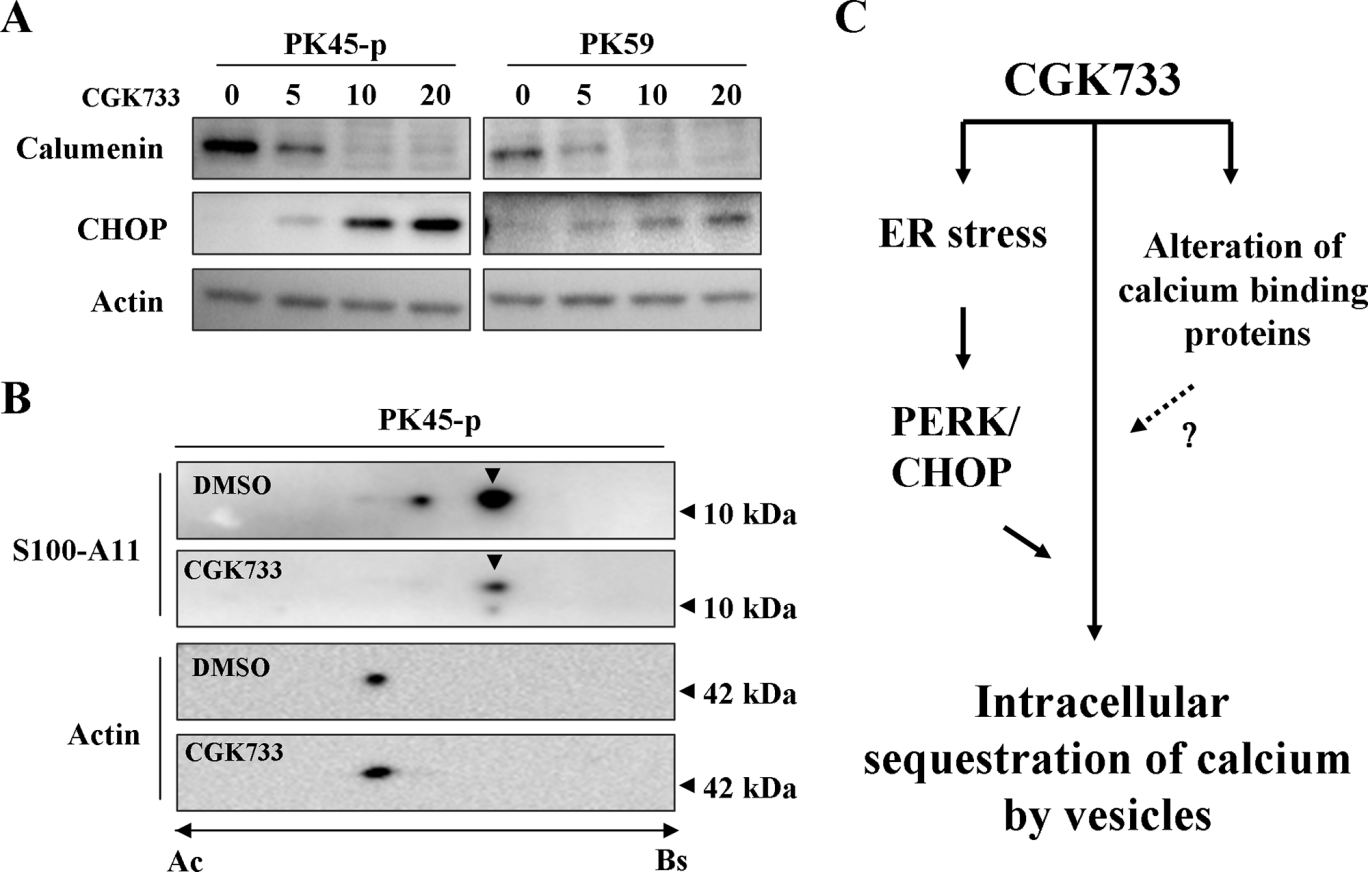
Alterations of calumenin and protein S100-A11 were induced by treatment with CGK733 **A.** The expressions of calumenin, protein S100-A11 and CHOP were detected by Western blotting after cells were treated with CGK733 for 6 h in a dose dependent manner. **B.** The isoforms of protein S100-A11 were detected by 2-D Western blotting after cells were exposed to 20 μM of CGK733. Arrow heads indicate the isoform shown in the 2-DE gel image in Figure 7A. **C.** Scheme summarizing the CGK733-induced signaling pathway in pancreatic cancer cells.

To study the mechanism of CGK733-induced vesicular calcium sequestration, comparative proteomic analysis was performed after cells were treated or not with CGK733 for 6 h. Three protein spots were shown in the 2-DE gel image to be down-regulated by CGK733 treatment for 6 h (Figure 6A). These protein spots were identified by LC-MS/MS as calumenin and protein S100-A11 (Figure 6B and Table 1). The down-regulation of calumenin expression and the post-translational modifications of S100-A11 were induced by CGK733 treatment for 6 h, as shown in the Western blot and 2-D Western blot assays, respectively (Figure 7A and 7B). Knockdown of CHOP did not recover the reduced calumenin induced by CGK733 treatment (data not shown). These results indicated that calumenin and S100-A11 might at least partially contribute to the CGK733-induced vesicular calcium sequestration.

**Table 1:** Identification of Ca^2+^ banding proteins

2-DE (*n* = 5)					LC-MS/MS					
Spot no.	Expected (Observed)		Average Intensity	*P* value (ANOVA)	Database Accession	Spectra	Distinct Peptides	MS/MS Search score	% AA Coverage	Protein Name
	MW (kDa)	*p*I								
**422**	46.0 (37.2)	4.55 (4.47)	0.47	> 0.001	O43852	20	9	124.11	34.6	Calumenin
**427**	46.0 (37.2)	4.55 (4.47)	0.58	> 0.001	O43852	12	9	132.42	36.5	Calumenin
**771**	12.0 (11.8)	7.10 (6.57)	0.76	> 0.05	P31949	20	2	33.74	25.7	Protein S100-A11

Summary of the 2-DE and LC-MS/MS analysis showing details of the three protein spots identified.

## DISCUSSION

Perturbation of intracellular Ca^2+^ compartmentalization has be shown to trigger either apoptotic or necrotic cell death [22]. We herein show that CGK733 induces cell death via a previously undescribed mechanism which is calcium-correlated, yet apoptosis- or necrosis-independent as well as being distinct from its cell cycle arrest activity. Since the observed effects include ER stress, vesiculation, intact nuclei and loss of cellular membrane in the process of death, we deduce that CGK733 may trigger cell death through a TNF-α-independent necrotic pathway or by inducing irreparable damage in the ER. Possibly, the functional abnormality or original structural damage of the ER is expected to become the leading cause for the production of massive vesicles containing calcium. Although the mitochondria are also able to buffer intracellular calcium, none of the evidence from proteomic analysis has shown any mitochondrial involvement in CGK733-induced calcium sequestration. This is the first report of such an effect, with no previous link between CGK733 and calcium cycling in the cell being reported in the literature.

The observed process following CGK733 administration, involves the initial formation of calcium-laden vesicles, which appear to be linked to the ER stress response through the PERK/CHOP signaling pathway, which subsequently brings about non-apoptotic/necrotic cell death. Depletion of calcium levels from the ER lumen, oxidative stress, impairment of protein transport from the ER to the Golgi, and/or accumulation of misfolded protein in the ER can result in ER stress. Activation of the PERK pathway, following ER stress, reduces the protein load within the ER by reducing protein translation. ER stress also upregulates protein chaperones, amino acid metabolism and redox homeostasis [23].

ER stress signals are transduced by three ER membrane sensors: inositol requiring element-1 (IRE-1), PERK and activating transcription factor 6 (ATF6) [24]. PERK activation initiates eukaryotic initiation factor 2α (eIF2α) kinase activity, which in turn activates the transcription factor CHOP through the activating transcription factor 4 (ATF4) [25, 26]. Activation of IRE-1 is involved in protein maturation and folding, as well as export and degradation of misfolded proteins through splicing of X-box binding protein 1 XBP1 [27–29] Activation of ATF6 increases the transcription of ER chaperones, such as 78 kDa glucose-regulated protein (Bip/Grp78) and heat shock protein 90 kDa beta member 1 (Grp94) [24, 30].

PERK (like IRE1) is a type-I transmembrane protein kinase found in the ER, involved in the transmission of stress signals in response to protein misfolding. The activation of PERK and concommitant upregulation of CHOP by CGK733 together with no change in either phospho (p)-IREα or Bip, indicates that CGK733-induced ER stress response is specifically correlated with the PERK/CHOP signaling pathway. Moreover, since the lumenal domains of both PERK and IRE1 can form stable complexes with the ER chaperone Bip [31], it can be inferred that the mechanism by which Bip is released from the PERK and IRE1 lumenal domains is not involved in this process.

We postulate that CGK733 simulates an as yet unclear subset of the stress signals of misfolded proteins, leading to the sequestration of cellular calcium into vesicles, which can be dismantled once the drug treatment is stopped and ER signalling is restored. Calcium sequestration into vesicles mimics the stress response and initiates cell death, although the observed process is reversible, such that Ca^2+^ can be released back into the cell and block the ensuing cell death. Further investigation demonstrated that this intracellular calcium sequestration in vesicles is not directly correlated to the subsequent cell death, as shown by the use of ionomycin (as well as CHOP siRNA), indicating that the CGK733-induced ER stress response is possibly a parallel downstream effect to the observed cell death. Overall, the various results indicate that the PERK/CHOP signaling pathway is directly involved in the observed calcium sequestration, but does not appear to contribute to the consequent non-apoptotic/necrotic cell death induced by treatment with CGK733.

Although this mechanism was initially observed in pancreatic cancer cell lines, similar tests were carried out on the immortalised, non-cancer cell lines NIH3T3 and HEK293 and these showed the same result, indicating that this process is not an artifact of carcinogenesis but can be undergone by all cell types. A number of attempts were also made to prepare rat pancreatic primary cultures but very few cells grew out of the tissue fragments, making them insufficient for such a test.

Interestingly, proteomic analysis indicated that calumenin and S100-A11 might be, at least partially, contributing to the CGK733-induced calcium sequestration. Both calumenin and protein S100-A11 are EF-hand Ca^2+^-binding proteins and commonly located in the ER [32, 33]. Calumenin interacts with ER proteins as a chaperone and it also functions in the vitamin K-dependent-carboxylation system [34, 35], but little is known about its effects on the calcium signaling pathways. S100 proteins play a mediator role of calcium-associated signal transduction through thier effects on protein phosphorylation, which gives them the ability to regulate ion channels [22, 33].

Calumenin, has recently been shown to be upregulated by ER stress and bring about a reduction in ER-initiated apoptosis [36]. In the case of S100-A11, no direct link between its calcium-related functions and ER stress is available in the literature but the fact that two protein spots were isolated in this study indicates that specific post-translational modifications might contribute to the ER stress response or the process of calcium sequestration in vesicles.

The data gathered on the action of CGK733 in pancreatic cancer presents an interesting therapeutic opportunity to push tumours which are already under heavy stress towards non-apoptotic/necrotic cell death. This reversible ER stress-mimicking process possesses the added benefit of being easily reversible once it is deemed evident that the tumour has remitted and treatment is stopped. Moreover it can be used in combinatorial treatments as has already been shown with Taxol to treat *Hepatitis B virus*-positive hepatocellular carcinoma cells which are resistant to Taxol alone [40].

## MATERIALS AND METHODS

### Cell culture

The pancreatic cancer cell lines PK45-p, PK59, PANC-1 and MIA-PaCa-2 were provided by the Institute of Development, Aging and Cancer at Tohoku University. BxPC-3 and AsPC-1 were purchased from the American Type Culture Collection (ATCC). BxPC-3, AsPC-1, PK45-p and PK59 were cultured in Roswell Park Memorial Institute 1640 medium (RPMI 1640, 05918, GIBCO, Billings, MT) while PANC-1 and MIAPaCa-2 were cultured in Dulbecco's Modified Eagle's medium (DMEM, 12100-046, GIBCO, Billings, MT), supplemented with 10% heat-inactivated fetal bovine serum (FBS, 26140-079, GIBCO, Billings, MT), and 2 mM L-glutamine and incubated at 37°C in a humidified incubator containing 5% CO_2_.

### Materials

Anti-GFP (sc-9996), S100-A11 (sc-98427), PARP-1 (sc-8007) and actin (sc-1616) antibodies were purchased from Santa Cruz Biotechnology Inc, Santa Cruz, CA. Anti-caspase (#9661) antibody was purchased from Cell Signaling Technology Inc, Boston, MA. Anti-Bip (ab21685) and calumenin (ab137019) antibodies were purchased from Abcam Inc, Cambridge MA. Anti-p-IREα (NB-100-2323) antibody was purchased from Novus Biologicals Inc, Littleton, CO. Control (sc-37007) and CHOP (sc-35437) siRNA were purchased from Santa Cruz Biotechnology Inc. CGK733 (sc202964), Ionomycin (sc-300835), z-VAD-fmk (sc-3067) and Necrostatin-1 (sc-200142) were purchased from Santa Cruz Biotechnology Inc. Cal-520 No wash Calcium Assay Kit was purchased from Abcam Inc. Apoptosis and Necrosis Detection Kit (EthD III) (PK-CA707-30018) was purchased from Promokine Inc, Heidelberg, Germany. EGTA (342-01314) was purchased from Dojindo, Kumamoto, Japan. Thapsigargin (209-1281) was purchased from Wako, Osaka, Japan.

### Western blot

The cells were suspended in lysis buffer (1% NP-40, 1 mM sodium vanadate, 1 mM PMSF, 50 mM Tris, 10 mM NaF, 10 mM EDTA, 165 mM NaCl, 10 μg/mL leupeptin, and 10 μg/mL aprotinin) on ice for 1 h [37, 38]. Fifteen micro grams of protein were resolved by 5–20% SDS-polyacrylamide gel (SuperSep Ace 194-15021, Wako, Osaka, Japan) and then transferred onto PVDF membrane (Immobilon-P, Millipore, Bedford, MA). The membrane was incubated with the primary antibody at 4°C overnight and then incubated with a horseradish peroxidase (HRP)-conjugated secondary antibody for 1 h at room temperature. The signals were detected with a chemiluminescent reagent (Immunostar 290-69904, Wako, Osaka, Japan).

### Pathological staining

Cells were cultured on coverslips in 12 well plates at a density of 1 × 10^5^ cells per well. Cells were washed with PBS and fixed with 4% paraformaldehyde for 15 min and permeabilized with 0.1% Triton X-100 for 15 min. Cells were then stained by hematoxylin and eosin (H&E) staining (to observe the cell structure), PA-Schiff staining (to observe the glycogen particles) and Oil red O staining (to observe the lipid droplets), respectively.

### Microscopy

Cells were cultured on coverslips in 12 well plates at a density of 1 × 10^5^ cells per well. The treated cells were then observed directly under bright field or stained by appropriate staining dyes following the provided protocols. The calcium ions were stained using a Cal-520 fluorescent reagent kit following the manufacturer's protocol (Abcam). Confocal images were obtained using Nikon Plan Apo 60X/1.40 objective, BZ-9000 series (BIOREVO) and BZ-II Viewer software (Keyence, Osaka, Japan) by an operator who was unaware of the experimental condition. [39]

### Transient transfection

Cells were incubated at 37°C in a CO_2_ incubator until the cells were 70% confluent. Cells were transfected with validated siRNA by following the manufacturer's siRNA Transfection Protocol (Santa Cruz Biotechnology).

### MTS viability assay

Cell viability was determined by CellTiter 96^®^ AQueous One Solution Cell Proliferation Assay (Promega, Madison, WI). An aliquot of 20 μL of the MTS dye was added to each well of the plate and incubated for a further 2 h. Optical density (OD) was read at 492 nm using a Model 550 reader (BIO-RAD, Hercules, CA). The experiments were individually performed three times.

### Two-dimensional gel electrophoresis (2-DE)

Isoelectric focusing (IEF) was performed in an IPGphor 3 IEF unit (GE Healthcare, Buckinghamshire, UK) on 11 cm, immobilized linear pH gradient 3–10 linear gradient IPG strips (Bio-Rad) at 50 μA/strip. Three hundred μg of protein was used for each 2-DE. Protein was mixed into rehydration buffer [8 M urea, 2% CHAPS, 0.01% bromophenol blue, 1.2% Destreak reagent (GE Healthcare)] and 0.5% IPG buffer (GE Healthcare) and loaded into the IPGphor strip holder (GE Healthcare). The strips were then focused by the following steps: rehydration for 10 h (no voltage applied); 0 to 500 V for 4 h; 500 to 1,000 V for 1 h; 1,000 to 8,000 V for 4 h; 8,000 V for 20 min; and the final phase of 500 V from 20,000 to 30,000 Vh. The IPG strips were equilibrated in equilibration buffer 1 (6 M urea, 0.5 M Tris-HCl pH 8.8, 30% glycerol, 2% SDS, 2% 2-ME) and in equilibration buffer 2 (6 M urea, 0.5 M Tris-HCl pH 8.8, 30% glycerol, 2% SDS, 2.5% iodoacetoamide) for 10 min each. The IPG strips were then transferred onto precast polyacrylamide gels with a linear concentration gradient of 4–20% (Bio-Rad) and run at 200 V for 1 h. The gels were fixed in 40% ethanol and 10% acetic acid for more than 2.5 h. The gels were then stained with See Pico™ (Benebiosis, Seoul, Korea) overnight. The stained gels were analyzed by using the ProXpress 2-D Proteomic Imaging System (PerkinElmer, Waltham, MA) and Progenesis Samespots software (Nonlinear, Newcastle, upon Tyne, UK) [39]. The criteria used by the SameSpot software to determine the difference in spot intensity were: spot area x stain intensity. Following the automatic calculation of ANOVA by the software a manual cut-off point of *p* < 0.05 and more than 1.7 fold change in intensity was applied.

### LC-MS/MS

The gel sections were destained by rinsing in 60% methanol, 0.05 M ammonium bicarbonate, and 5 mM DTT three times for 15 min and rinsed twice in 50% methanol, 0.05 M ammonium bicarbonate, and 5 mM DTT for 10 min. The gel sections were dehydrated twice in 100% acetonitrile (ACN) for 30 min. Enzyme digestion was performed with 10 μg/mL sequencing-grade-modified trypsin (Promega) in 30% ACN, 0.05 M ammonium bicarbonate, and 5 mM DTT at 30°C for 16 h. The lyophilization was performed by using a Labconco Lyph-lock 1 L Model 77400 (Labconco, Kansas, MO) for 6 h. Protein peptides were dissolved in 15 μL of 0.1% formic acid for later analysis by a liquid chromatography tandem mass spectrometry (LC-MS/MS) system (Agilent 1100 LC-MSD Trap XCT; Agilent Technologies, Palo Alto, CA). The HPLC column used was a Zorbax 300 SB-C18 (Agilent Technology) of dimensions = 3.5 μm, 15 mm × 75 mm; using mobile phases = Solvent A: 0.1% formic acid and Solvent B: CH_3_CN in 0.1% formic acid; with a gradient = 0–5 min 2% B, 78 min 60% B; a flow rate = 0.3 μl/min; stop time = 78 min. MS instrument settings were: capillary voltage at – 1600 V; dry gas at 5.0 cl/min, dry temperature of 500°C, and a relative collision energy of 1.15 V. The scanning range was from 286 to 2200 m/z and a threshold of 100,000. Each peptide scan picked 4 precursor ions (threshold abs. = 10000). The mass window for precursor ion selection used was between 286 to 2200 m/s to reduce the background intensity. The isolated masses were excluded after 2 spectra and released after 0.5 min.

MS data was analyzed by the Agilent Spectrum Mill MS proteomics workbench (ver. Rev B.04.00.141) against the Swiss-Prot protein database (http://kr.expasy.org/sprot/). The number of sequences in the Swiss -Prot database used for the Spectrum Mill analyses was 540546 and the download date was 17/7/2013. The criteria for positive identification of proteins were set up: filter by protein score > 10.0, and filter peptide by score > 8, percentage scored peak intensity (% SPI) > 70 [38, 39].

## SUPPLEMENTARY FIGURES


