# Supplementary material for: PERK/CHOP contributes to the CGK733-induced vesicular calcium sequestration which is accompanied by non-apoptotic cell death
Source: Oncotarget. 2015 Jul 10;6(28):25252–65. doi: 10.18632/oncotarget.4487 (PMC4694829; doi:10.18632/oncotarget.4487)
Supplement: Supplementary file 1 [file oncotarget-06-25252-s001.pdf]

SUPPLEMENTARY FIGURES

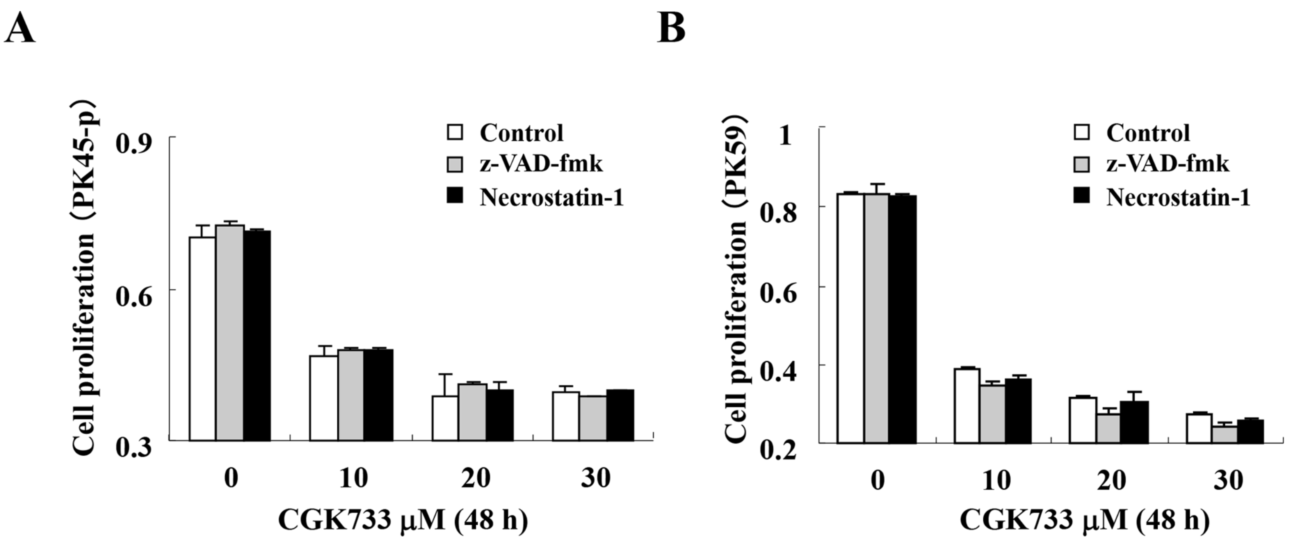

Supplementary Figure S1: GK733-induced cell death is independent of apoptosis or necroptosis. A. and **B.** PK45-p and PK59 cells were treated with CGK733 for 48 h in a dose dependent manner after cells were pre-treated with either 100 μM of z-VAD-fmk or 50 μM of Necrostatin-1.

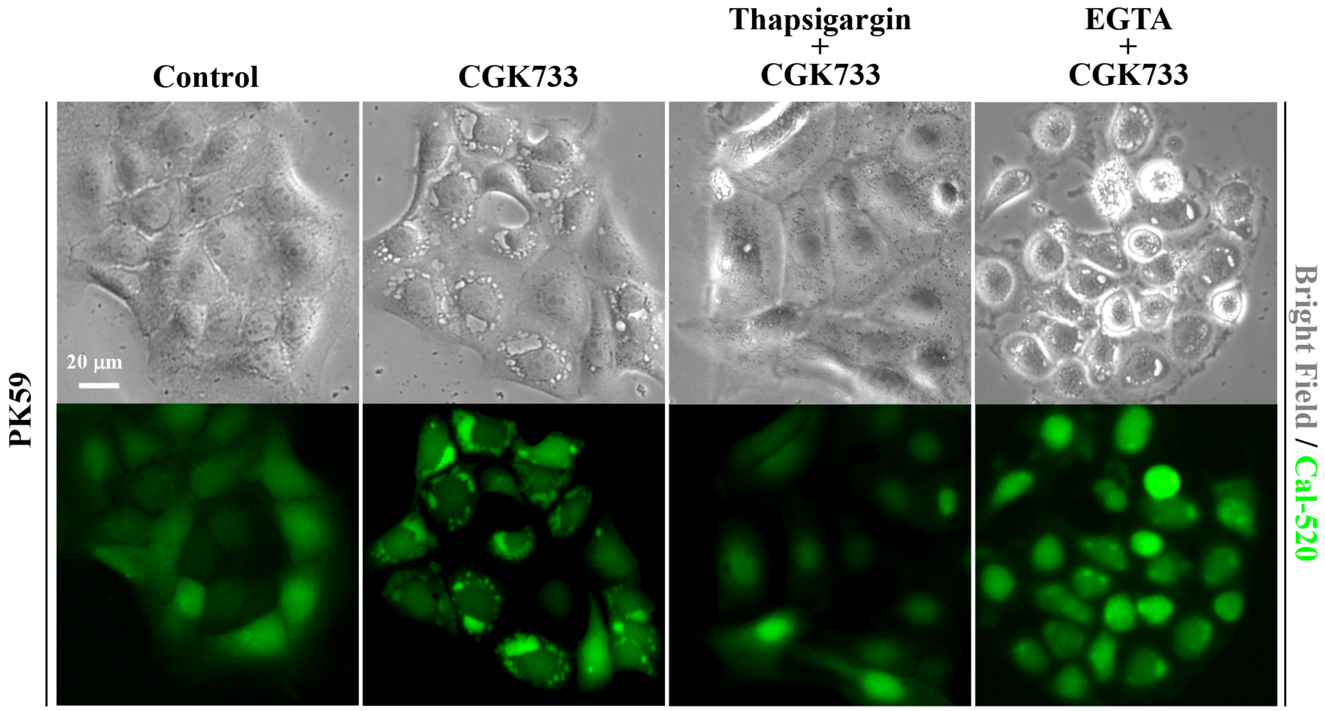

Supplementary Figure S2: Thapsigargin inhibits CGK733-induced vesicular calcium sequestration in PK59 cells. PK59 cells were treated with 20 μM of CGK733 alone for 6 h or pre-treated with either 1 μM of Thapsigargin or 10 mM of EGTA.
